# Shift work and risk of incident dementia: a study of two population-based cohorts

**DOI:** 10.1007/s10654-018-0430-8

**Published:** 2018-08-03

**Authors:** Kathleen Bokenberger, Arvid Sjölander, Anna K. Dahl Aslan, Ida K. Karlsson, Torbjörn Åkerstedt, Nancy L. Pedersen

**Affiliations:** 1 Department of Medical Epidemiology and Biostatistics, Karolinska Institutet, 17177 Stockholm, Sweden; 2 School of Health and Welfare, Institute of Gerontology, Jönköping University, 55111 Jönköping, Sweden; 3 Stress Research Institute, Stockholm University, 10691 Stockholm, Sweden; 4 Department of Clinical Neuroscience, Karolinska Institutet, 17177 Stockholm, Sweden; 5 Department of Psychology, University of Southern California, Los Angeles, CA 90089-1061 USA

**Keywords:** Shift work, Night shift work, Dementia incidence, Epidemiology, Prospective cohort

## Abstract

This study aimed to investigate the association between shift work and incident dementia in two population-based cohorts from the Swedish Twin Registry (STR). The STR-1973 sample included 13,283 participants born 1926–1943 who received a mailed questionnaire in 1973 that asked about status (ever/never) and duration (years) of shift work employment. The Screening Across the Lifespan Twin (SALT) sample included 41,199 participants born 1900–1958 who participated in a telephone interview in 1998–2002 that asked about night work status and duration. Dementia diagnoses came from Swedish patient registers. Cox proportional-hazards regression was used to estimate hazard ratios (HR) with 95% confidence intervals (CI). Potential confounders such as age, sex, education, diabetes, cardiovascular disease and stroke were included in adjusted models. In genotyped subsamples (n = 2977 in STR-1973; n = 10,366 in SALT), *APOE* ε4 status was considered in models. A total of 983 (7.4%) and 1979 (4.8%) dementia cases were identified after a median of 41.2 and 14.1 years follow-up in the STR-1973 and SALT sample, respectively. Ever shift work (HR 1.36, 95% CI 1.15–1.60) and night work (HR 1.12, 95% CI 1.01–1.23) were associated with higher dementia incidence. Modest dose-response associations were observed, where longer duration shift work and night work predicted increased dementia risk. Among *APOE* ε4 carriers, individuals exposed to ≥ 20 years of shift work and night work had increased dementia risk compared to day workers. Findings indicate that shift work, including night shift work, compared to non-shift jobs is associated with increased dementia incidence. Confirmation of findings is needed.

**Electronic supplementary material:**

The online version of this article (10.1007/s10654-018-0430-8) contains supplementary material, which is available to authorized users.

## Introduction

Shift work, which may include working at night, has been demonstrated to disrupt biological circadian rhythms and the sleep and wake cycle [1, 2]. Full circadian adjustment is seldom accomplished in night shift workers due to factors interfering with the circadian system such as daylight during day sleep [2]. Inability in synchronizing the biological clock with one’s shift work schedule can manifest as curtailed sleep, increased fatigue, unhealthy lifestyle, and cognitive impairment [2, 3].

Previous research suggests a connection between sleep deprivation and pathological signs of Alzheimer’s disease (AD), a dementia subtype for which old age and presence of the apolipoprotein E (*APOE*) ε4 allele are among the strongest risk factors [4]. A cross-sectional study found that adults who reported shorter sleep durations had greater concentrations of amyloid-β in the brain [5]. In a similar vein, chronic sleep restriction for 21 days was correlated with elevated amyloid-β plaque accumulation in mice [6]. Further, greater sleep disturbance [7] and short time in bed [8] were associated with increased dementia incidence.

A recent cohort study of about 18,000 nurses exploring the effect of shift work on cause-specific mortality showed an association between rotating (alternating between day, evening or night shifts) and evening shift work with mortality from AD and dementia [9]. Another study that followed 4766 Danish men did not observe an association between ever shift work, defined as shift work including night work, and incidence of dementia [10]. As these are the only studies, to our knowledge, that have investigated the association between shift work and subsequent dementia risk or mortality, more prospective research is needed to elucidate the impact of shift work, with or without night work, in relation to dementia risk.

The present study aims to examine the association between shift work and incident dementia in two population-based cohorts from the Swedish Twin Registry (STR). For this purpose, we used one cohort with information on the number of years with ever shift work, and another cohort that with information on the number of years with night work.

## Methods

### Participants

**Fig. 1:**
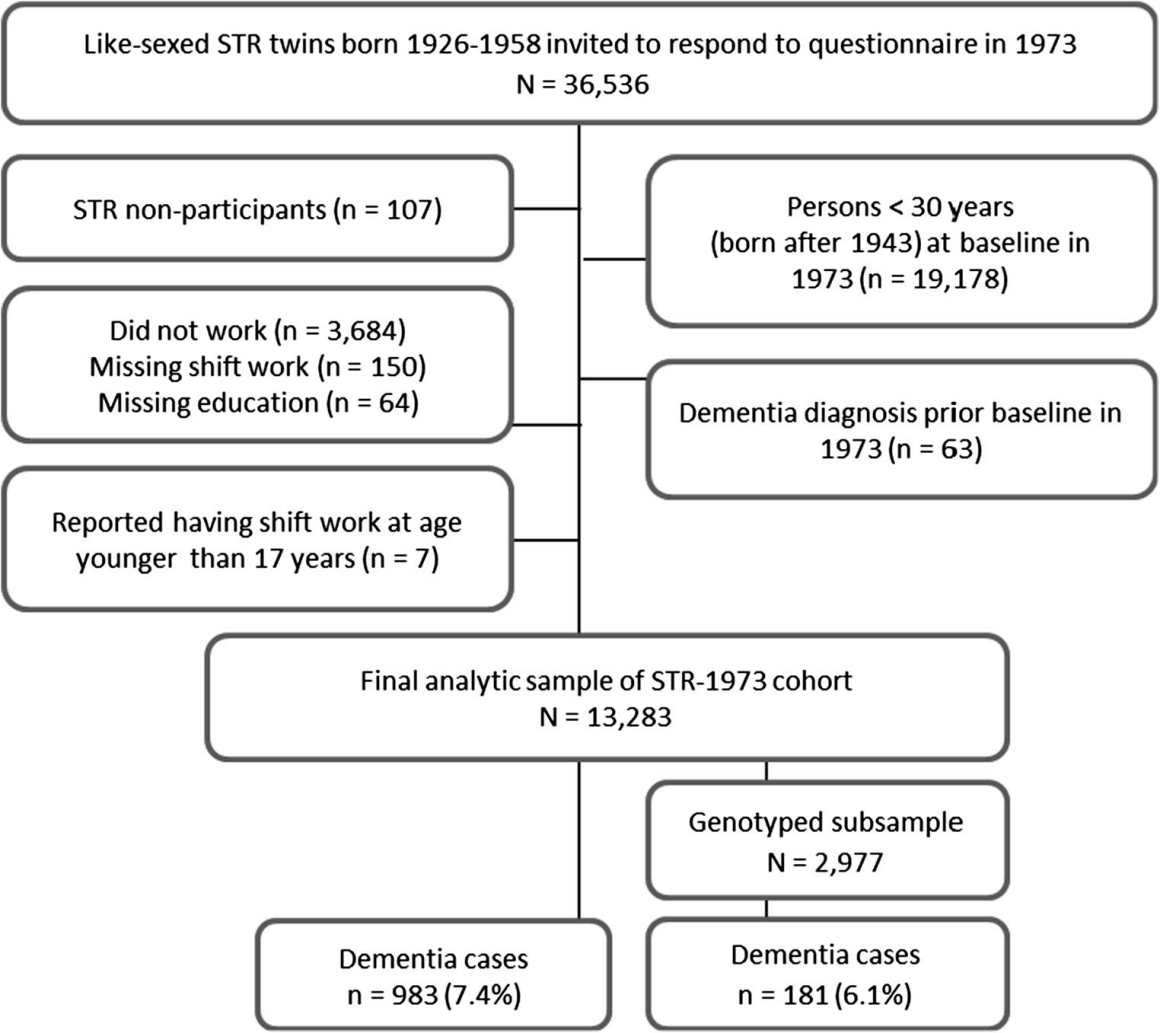
Flow chart of stepwise participant selection in the STR-1973 cohort

**Fig. 2:**
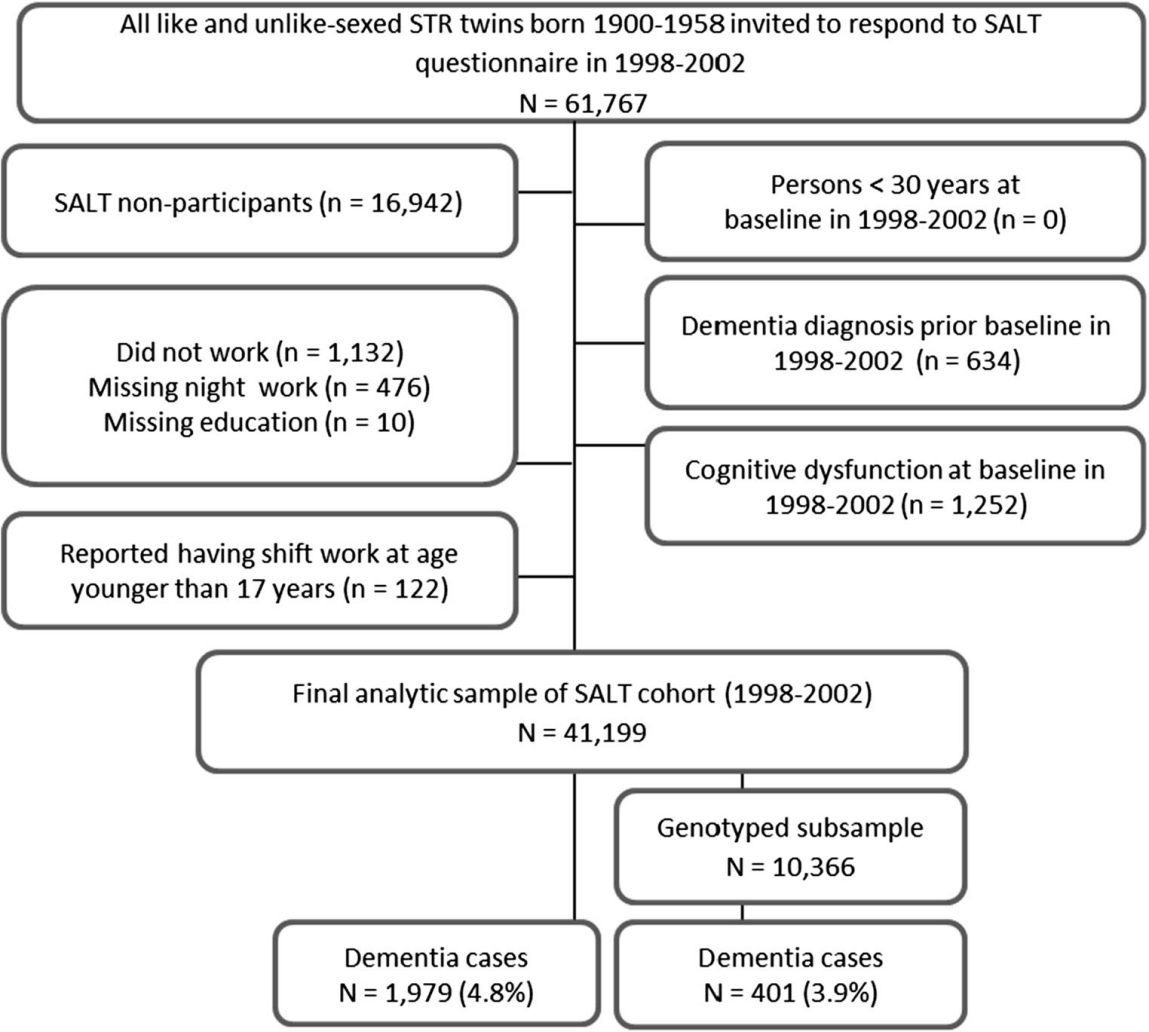
Flow chart of stepwise participant selection in the SALT cohort

The STR is the largest twin registry in the world and includes all twins born in Sweden since 1886 [11, 12]. Two STR cohorts were used: a cohort born 1926–1958 who were mailed a questionnaire in 1973 (hereinafter referred to as the STR-1973 cohort), and a cohort born 1958 or earlier who were invited to participate in a telephone-administered interview in the Screening Across the Lifespan Twin (SALT) study in 1998–2002 (hereinafter referred to as the SALT cohort). STR-based studies have been described in detail previously [11, 13]. Both cohorts were asked about ifestyle-related behaviors, disease history, and occupational information such as exposure to shift work. Flow charts of the stepwise participant selection process from each cohort are provided in Figs. 1 and 2.

SALT additionally administered a cognitive screening that included a mental status examination known as the TELE [14]. Together with health and daily functioning assessments and informant interviews for participants who performed poorly on the mental status examination, an ordinal cognitive status scale was created (0 = no cognitive dysfunction, 1 = minor errors, 2 = poor cognitive performance but no confirmation of interference with daily functioning, 3 = cognitive dysfunction sufficient to interfere with daily function) [15]. Since it was possible that those with baseline cognitive dysfunction (score of 3) were preclinical dementia cases, these individuals (n = 1252) were excluded from the SALT sample.

Both STR-1973 and SALT samples were restricted to individuals at least 30 years of age at the time of each respective baseline, 1973 and 1998–2002. The rationale for this restriction was to include persons who had sufficient duration of job experience at the time of responding to the questionnaire. Thus, participants were born 1926 to 1943 in the STR-1973 sample, and born 1900 to 1958 in the SALT sample. Those who reported having shift work at an age younger than 17 years were excluded as we suspected these were invalid responses. Both samples also excluded persons diagnosed with dementia prior toand at baseline to minimize risk of reverse causality. Individuals who did not work were excluded. In SALT, those who did not work (n = 1132) included housewives/men who never had jobs (n = 303) but not housewives/men who ever had jobs. Housewife/men status was not available in the STR-1973 questionnaire. After exclusions, the STR-1973 sample included 13,283 participants, and the SALT sample included 41,199 participants. As both cohorts came from the STR, there is an overlap of 8904 participants between the two samples.

Additionally, subsamples from STR-1973 (n = 2977) and SALT (n = 10,366) were genotyped as part of the TwinGene study between 2004 and 2008 [12], and thus had data on the *APOE* genotype.

All samples were followed up until the date of dementia diagnosis, death, or end of study period on 31 December 2014.

### Incident dementia

Incident dementia diagnoses were identified according to the International Classification of Disease [ICD] versions 8, 9, and 10 codes and Anatomical Therapeutic Chemical Classification System [ATC] codes. ICD codes were obtained from the National Patient Register (NPR), including both inpatient records since 1967 (complete coverage since 1987) and outpatient records since 2001, and the Cause of Death Register (CDR), which includes death dates and underlying and contributing death causes since 1952. ATC codes were obtained from the Prescription Drug Register (PDR), which contains records of prescribed medications since 2005, with individuals with a prescription for anti-dementia medication defined as a case. Diagnoses were based on the following codes: 290, 293.0, 293.1 in ICD-8; 290.0, 290.1, 331.0, 290.4, 290.8, 290.9, 294.1, 331.1, 331.2, 331.9 in ICD-9; G30, F01, F02, F03, F05.1, G31.1, G31.8A in ICD-10; ATC codes classified under N06D for anti-dementia drugs.

### Shift work measures

In 1973, STR-1973 participants were presented with the item concerning shift work: “Do you work or have you worked shifts?” Response alternatives were No or Yes. Upon responding Yes, the respondent was asked to specify the time spend working shifts in years.

If one responded Yes, the respondent was asked to specify for how long time in years.

In 1998–2002, SALT participants were asked an open-ended question concerning night work: “During about how many years have you had working hours that meant that you at least sometimes worked at night?” No night shift work was recorded as 0 by the interviewer. For both shift work and night work measures, an ever shift work history measure (ever/never) was derived. Cumulative exposure duration measures for shift work and night work were assessed as a categorical variables (none, 1–9 years, 10–19 years, 20 or more years) as was similarly done in earlier studies examining shift work duration [16–18], and as continuous cubic spline variables with 3 knots (1 year, 10 years, and 20 years) and 4 knots (1, 5, 10, 20 years) selected a priori based on comparable groupings of work duration in previous work [16–18]. Those who responded No to having worked shifts or nights are referred to as “dayworkers” throughout this paper.

### Covariates

Demographic information on age, sex, and education (grouped as 7 years or less versus more than 7 years of education) came from the STR. Data on hypertension (yes/no) came from the SALT questionnaire. Data on diagnoses for diabetes of all types, cardiovascular disease (CVD) and stroke came from the SALT questionnaire and via ICD codes from the NPR. CVD included angina pectoris, atherosclerosis, cardiac valve murmurs, claudication, heart failure, high cholesterol, ischemic heart disease, irregular heartbeat, myocardial infarction, narrowing of the carotid arteries, thrombosis, and transient ischemic attacks. Stroke included ischemic or hemorrhagic stroke. Information on hypnotics usage (grouped as yes/no) was based on items on sleeping pill and tranquilizer use from the 1973 questionnaire, as well as on ATC codes classified under N05C for hypnotics and sedatives based on information from SALT and the PDR. In SALT, a subsample of participants 65 years and older (N = 9853) were asked about sleep-related experiences during the last 6 months. Sleep parameters included time in bed (≤ 6, 6–9, > 9 h), early rising time (rising before 8:00 AM vs later rising), and early bed time (bed time 11:00 PM or later vs earlier bed time). Measures for sleep quality (n = 4683), non-restorative sleep (n = 4703), and heavy snoring (yes/no) (n = 4197) were available for a smaller subset of this older study population.

Genetic covariates included *APOE* ε4 status (dichotomized as carrier and non-carrier of the ε4 allele) in the TwinGene subsample who were genotyped using Illumina OmniExpress. Genotype imputation was performed based on the 1000 Genomes Project [19].

### Statistical analyses

Cox proportional-hazards models were used to estimate hazard ratios (HR) for dementia with 95% confidence intervals (CI). Cluster-robust standard errors were used, which correct for the dependence between twins from the same pair [20]. Age was used as the underlying timescale. Proportional hazards assumptions were justified based on Schoenfeld residual tests and log-log survival plots.

Two sets of Cox analyses (one based on the STR-1973 sample, the other on the SALT sample) were performed. The first set of analyses entailed examining shift work status (no shift work as the reference group) and shift work duration (0 years of shift work as the reference category) as exposure variables in separate Cox models with t0 (baseline) in 1973 for the STR-1973 sample. The second set of analyses looked at night shift work status (no night shift work as the reference group) and night shift work duration (0 years of night shift work as the reference category) as exposure variables in separate Cox models with t0 in 1998–2002 for the SALT sample. Final models were adjusted for age, sex, education, diabetes, CVD and stroke. Diabetes, CVD and stroke that occurred prior to a dementia diagnosis were treated as time-varying covariates, while all other covariates were treated as fixed. In both sets of analyses, the covariate for hypnotics use was considered but dropped from the final models since HR estimates were generally unaffected by this covariate.

In addition to treating shift work/night work duration variables as categorical, we also transformed shift work/night work duration variables into restricted cubic spline variables with three knots at 1, 10, and 20 years in Cox models.

Next we performed four sensitivity analyses, all fully adjusted. The first 3 sensitivity analyses assessed if different approaches to treating the exposure would influence findings. In contrast to performing two separate analyses as described above, the following analyses bring together shift work information from both data sources into one model. Inferences drawn from these sensitivity analyses may therefore only be generalized to any-type shift work and not night shift work specifically. The last sensitivity analysis was based on samples selected on different criteria determined a priori.

- In the first sensitivity analysis, t0 in 1973, and shift work was treated as a time-varying exposure such that shift work status, determined from the STR-1973 questionnaire, may change in 1998–2002 depending on the shift work status information provided when SALT began. In this scenario, those who were only in STR-1973 and not in SALT had a fixed shift work variable during the entire follow-up course.
- In the second sensitivity analysis, t0 in 1998–2002 (SALT baseline) and shift work information came from both STR-1973 and SALT. For those who participated in both STR-1973 and SALT, only shift work duration data from SALT was considered based on the assumption that the duration reported in SALT covered the duration reported in STR-1973. For those who participated in only one of the studies, shift work duration data came from whichever study they had participated in.
- The third sensitivity analysis was similar to the second sensitivity analysis. The difference is that for individuals who participated in both STR-1973 and SALT, shift work duration was based on the sum of the shift work durations reported in both data sources.
- The final sensitivity analysis involved restricting the sample to those born after 1925 (N = 37,270) so as to make the SALT sample more comparable to the STR sample born 1926–1958.

We also analyzed subsamples of genotyped individuals using Cox models to estimate the association between shift work and incident dementia while stratifying on *APOE* ε4 carrier status. Additionally, within-pair analyses were performed based on each of the cohorts using stratified Cox models, in which each twin pair is treated as a separate stratum, thereby implicitly controlling for all measured and unmeasured characteristics that twins from the same pair have in common. These models were fitted both to all twin pairs, and to the subset of monozygotic twin pairs. A substantial change in HR estimates in conditional within-pair models compared to unconditional full sample models would suggest familial factors confounding the association between shift work and dementia. Familial factors include genetic or shared environmental influences. Data management was performed in SAS 9.4 and data analyses in STATA 14.1.

## Results

### Participant characteristics

STR-1973 participants had a mean age of 37.8 years (standard deviation, SD 5.4) at baseline and were followed up for a median of 41.2 years. Shift workers comprised 17.0% of the sample and had a median shift work exposure of 4 years (range 1 to 30). A total of 983 cases of dementia (7.4%) were identified during the course of follow-up. SALT participants had a mean age of 58.5 years (SD 10.4) at baseline and were followed up for a median of 14.1 years. Night workers represented 30.1% of the SALT sample and had a median of 8 years night work exposure (range 1 to 76). A total of 1979 cases of dementia (4.8%) were identified.

Table 1 summarizes characteristics of participants in both STR-1973 and SALT samples. More men than women worked shifts. Proportions of diabetes and CVD or stroke were slightly higher among shift workers compared to dayworkers.

**Table 1:** Characteristics of participants in STR-1973 and SALT samples

	STR-1973 sample Baseline in 1973			SALT sample Baseline in 1998–2002		
	All (N = 13,283)	No shiftwork history (n = 11,025)	Shiftwork history (n = 2258)	All (N = 41,199)	No night work history (n = 28,800)	Night work history (n = 12,399)
Any shift work history	(N = 13,290)					
None	11,025 (83.0%)	11,025 (100%)	–	–	–	–
1–9 years	1687 (12.7%)	0 (0%)	1687 (74.7%)			
10–19 years	468 (3.5%)	0 (0%)	468 (20.7%)			
20+ years	103 (0.8%)	0 (0%)	103 (4.6%)			
Night work history						
None	–	–	–	28,800 (69.9%)	28,800 (100%)	–
1–9 years				6513 (15.8%)	0 (0%)	6513 (52.5%)
10–19 years				2764 (6.7%)	0 (0%)	2764 (22.3%)
20+ years				3122 (7.6%)	0 (0%)	3122 (25.2%)
Baseline age (M ± SD; Range)	37.8 ± 5.4; 30.0–47.0	37.8 ± 5.4; 30.0–47.0	37.7 ± 5.4; 30.0–47.0	58.1 ± 10.1; 41.0–99.0	58.4 ± 10.1; 41.0–99.0	57.2 ± 10.0; 41.0–98.0
Sex (N %)						
Male	6445 (48.5%)	4861 (44.1%)	1584 (70.1%)	19,249 (46.7%)	11,967 (41.5%)	7282 (58.7%)
Female	6838 (51.5%)	6164 (55.9%)	674 (29.9%)	21,950 (53.3%)	16,833 (58.5%)	5117 (41.3%)
Highest educational attainment						
≤ 7 years	6271 (47.2%)	5035 (45.7%)	1236 (54.7%)	13,707 (33.3%)	9819 (34.1%)	3888 (31.4%)
> 7 years	7012 (52.8%)	5990 (54.3%)	1022 (45.3%)	27,492 (66.7%)	18,981 (65.9%)	8511 (68.6%)
Hypnotics use						
No	8386 (63.1%)	6943 (63.0%)	1443 (63.9%)	28,623 (69.5%)	20,055 (69.6%)	8568 (69.1%)
Yes	4897 (36.9%)	4082 (37.0%)	815 (36.1%)	12,576 (30.5%)	8745 (30.4%)	3831 (30.9%)
Dementia						
No	12,300 (92.6%)	10,236 (92.8%)	2064 (91.4%)	39,220 (95.2%)	27,383 (95.1%)	11,837 (95.5%)
Yes	983 (7.4%)	789 (7.2%)	194 (8.6%)	1979 (4.8%)	1417 (4.9%)	562 (4.5%)
Diabetes						
No	11,486 (86.5%)	9582 (86.9%)	1904 (84.3%)	36,994 (89.8%)	25,984 (90.2%)	11,010 (88.8%)
Yes	1797 (13.5%)	1443 (13.1%)	354 (15.7%)	4205 (10.2%)	2816 (9.8%)	1389 (11.2%)
CVD or stroke						
No	8799 (66.2%)	7329 (66.5%)	1470 (65.1%)	27,070 (65.7%)	19,138 (69.5%)	7932 (64.0%)
Yes	4484 (33.7%)	3696 (33.5%)	788 (34.9%)	14,129 (34.3%)	9662 (33.5%)	4467 (36.0%)
*APOE* ε4 carrier	(N = 2977)	(n = 2487)	(n = 490)	(N = 10,366)	(n = 7354)	(n = 3012)
No	2046 (68.7%)	1702 (68.4%)	344 (70.2%)	7226 (69.7%)	5090 (69.2%)	2136 (70.9%)
Yes	931 (31.3)	785 (31.6%)	146 (29.8%)	3140 (30.3)	2264 (30.8%)	876 (29.1%)

*APOE* ε4 information was available in smaller genotyped subsamples of STR-1973 (N = 2977) and SALT (N = 10,366)

M, mean; SD, standard deviation; GRS, genetic risk score

A few cohort differences were noted. The proportion of individuals who were exposed to 20 years or more of shift work was smaller in the STR-1973 sample (4.6%) compared to the SALT sample (25.2%). This, however, could be explained by the fact that SALT participants were on average about 20 years older at baseline compared to STR-1973 participants and were therefore more likely to have accumulated more exposure time. SALT had a larger proportion of higher educated individuals, likely due to our restriction criteria whereby the youngest individuals were born 1958 in SALT and only 1943 in STR-1973.

### Association between shift work and incident dementia

Table 2 reports the results from the main Cox analyses for each cohort. In the multivariable-adjusted models, having shift work versus day work, as well as night work versus day work, were associated with higher rates of dementia. Models separately adjusted for diabetes and for CVD and stroke yielded similar estimates to the hazard ratios of 1.36 and 1.12 from the fully adjusted models (Model 4 in Table 2). In sensitivity analyses, hypertension was adjusted for in subsamples in the SALT cohort (N = 41,190) and STR-1973 cohort (N = 9248) that had information on hypertensive status. However, results were largely unaffected for ever shift work (HR 1.36, 95% CI 1.12–1.66) and for ever night work (HR 1.12, 95% CI 1.02–1.24). Comparison of descriptive characteristics showed that, apart from having a higher proportion of CVD or stroke (41.5%), the STR-1973 subsample with hypertensive data (N = 9248) was similar to the full STR-1973 sample (N = 13,283).

**Table 2:** Associations of shift work status and shift work duration with incident dementia based on the STR-1973 cohort (N = 13,283) and SALT cohort (N = 41,199)

	Model 1		Model 2		Model 3		Model 4 (Final)	
	HR	95% CI	HR	95% CI	HR	95% CI	HR	95% CI
Shift work in STR-1973 cohort (N = 13,283)								
No	1.00	–	1.00	–	1.00	–	1.00	–
Yes	1.40	1.19–1.65	1.41	1.19–1.66	1.40	1.18–1.65	1.36	1.15–1.60
Shift work duration in STR-1973 cohort (N = 13,283)								
None	1.00	–	1.00	–	1.00	–	1.00	–
1–9 years	1.37	1.14–1.66	1.37	1.13–1.66	1.37	1.13–1.65	1.32	1.09–1.60
10–19 years	1.67	1.26–2.23	1.69	1.26–2.26	1.67	1.25–2.24	1.53	1.14–2.06
≥ 20 years	0.92	0.50–1.72	0.93	0.50–1.73	0.90	0.48–1.68	1.12	0.58–2.15
Night work in SALT cohort (N = 41,199)								
No	1.00	–	1.00	–	1.00	–	1.00	–
Yes	1.13	1.02–1.25	1.12	1.02–1.24	1.12	1.01–1.24	1.12	1.01–1.23
Night work duration in SALT cohort (N = 41,199)								
None	1.00	–	1.00	–	1.00	–	1.00	–
1–9 years	1.10	0.97–1.25	1.10	0.97–1.25	1.10	0.96–1.25	1.13	0.99–1.28
10–19 years	1.14	0.94–1.37	1.13	0.94–1.37	1.13	0.94–1.37	1.06	0.88–1.28
≥ 20 years	1.17	0.99–1.37	1.16	0.98–1.37	1.15	0.98–1.36	1.14	0.96–1.34

Model 1 adjusted for age (age as the timescale). Model 2 adjusted for age, sex and education. Model 3 adjusted for age, sex, education, and hypnotics use. Model 4 adjusted for age, sex, education, diabetes, cardiovascular disease and stroke

HR, hazard ratio; CI, confidence interval

In subsamples of the SALT cohort 65 years and older with data on sleep items and with no prevalent dementia or cognitive dysfunction, we controlled for time in bed, rising time, and bedtime, sleep quality, non-restorative sleep, and heavy snoring. Adjustment for these sleep parameters did not influence estimates of association between shift work and dementia (results not shown).

There appeared to be a dose-response association between night shift work duration and dementia risk in the SALT sample, wherein longer night shift work duration predicted increased dementia rates (*p*  =  0.02 for linear trend).

Presence of a dose-response association between shift work and dementia in the STR-1973 sample was less clear, though the *p*-value for linear trend was highly significant (*p*  =  < 0.001). While 1–9 years and 10–19 years of shift work was predictive of higher dementia rates compared to no shift work, having 20 years or more of shift work was not significantly predictive of dementia, perhaps due to a smaller sample having extensive shift work history.

**Fig. 3:**
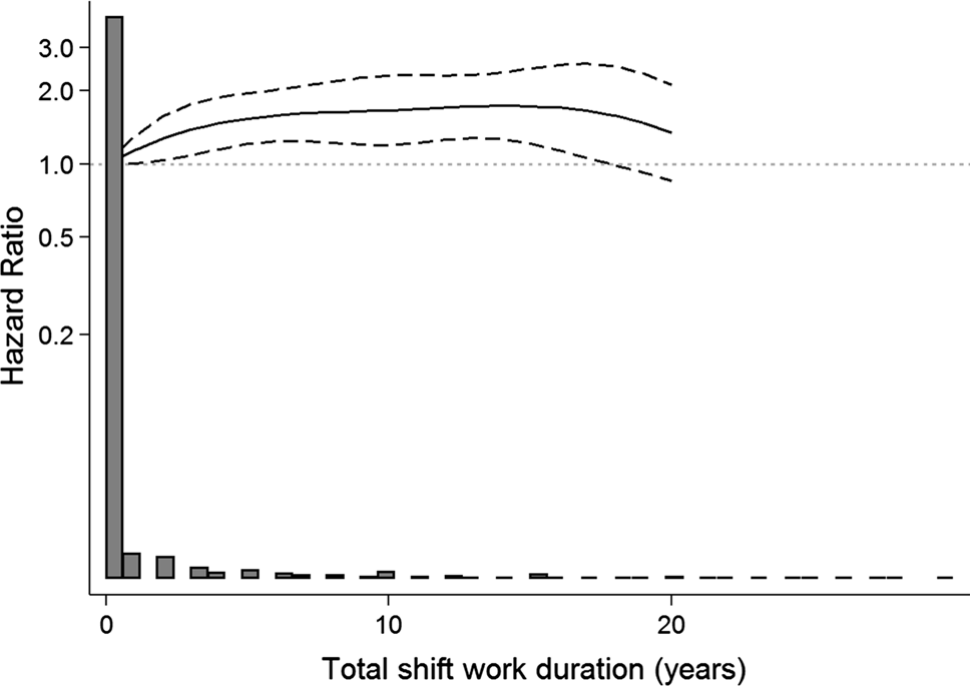
Hazard ratios of the association between shift work duration and incident dementia plotted on a log scale based on the STR-1973 sample (N = 13,283)

**Fig. 4:**
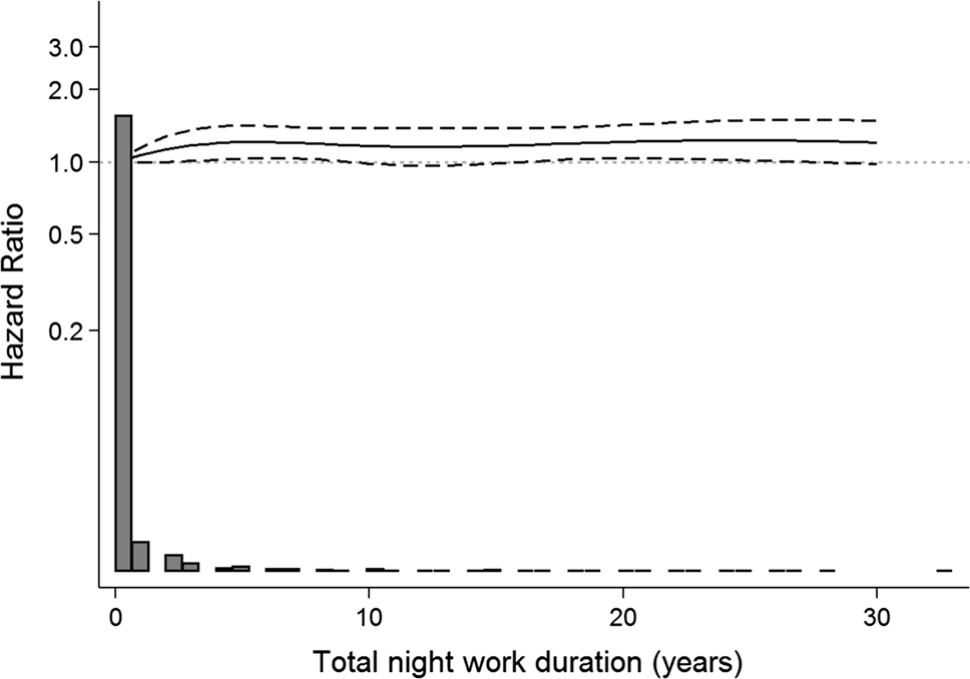
Hazard ratios of the association between night shift work duration and incident dementia plotted on a log scale based on the SALT sample (N = 41,199)

Spline models, regardless of the placement or number of knots described earlier in the methods, showed similar trends for the overall association between incident dementia and shift work (Fig. 3) and night work (Fig. 4). In both Figs. 3 and 4, in which we present splines with 3 knots, the association became slightly stronger with extended duration of shift work/night work. In Fig. 3, a slight dip in the hazard ratio was observed at approximately 18 to 20 years of shift work exposure.

The four aforementioned sensitivity analyses yielded stable findings that did not depart from the main analyses findings (Appendix A of Electronic Supplementary MAterial), indicating that our results for the association of shift work/night work with dementia risk are robust.

Within-pair analyses, that is, analyses conditioned on twin pair membership, were performed. These analyses were done separately in a subset of all twin pairs with known zygosity and in a subset of monozygotic pairs only. Compared to the unpaired analysis results, within-pair analysis of the SALT sample including both monozygotic and dizygotic twins showed similar, but slightly larger, magnitudes of associations for dementia risk with ever shift work (HR 1.28, 95% CI 1.00–1.64) and with ever night shift work (HR 1.28, 95% CI 1.00–1.64). Among monozygotic pairs only, there appeared to be a trend of a modest attenuation of associations between incident dementia with ever shift work (HR 1.12, 95% CI 0.58–2.15) and with ever night shift work (HR 1.01, 95% CI 0.55–1.85), though the associations were not significant. There was no obvious dose-response relationship observed from within-pair analyses of the association between incident dementia with duration of shift work and night work among all twin pairs and among MZ twin pairs (Appendix B of Electronic Supplementary MAterial).

### Analyses of genotyped subsamples

We explored interactions of shift work/night work with *APOE* ε4 (Table 3) by stratifying on *APOE* ε4 status. *APOE* ε4 appeared to modify the association between night work and dementia risk, but not the association between shift work and subsequent dementia. Among carriers of the ε4 allele, the association was strongest for individuals exposed to 20+ years of shift work or night work compared to individuals never exposed to shift work/night work. However, the trend test p-value for the association of dementia risk with shift work duration (*p* =  .78) and night work duration (*p* = 0.21) among the ε4 carrier group was not significant.

**Table 3:** Findings from interaction analyses between shift work/night work and *APOE* ε4 with respect to incident dementia

	Shift work status in STR sample (N = 2977) HR (95% CI)	
	*APOE* ε4 non-carrier (n = 2046)	*APOE* ε4 carrier (n = 931)
Shift work		
No	1.00	1.00
Yes	1.49 (0.92–2.41)	1.45 (0.85–2.49)
Shift work duration		
None	1.00	1.00
1–9 years	1.29 (0.70–2.37)	1.48 (0.79–2.76)
10–19 years	1.88 (0.94–3.77)	1.19 (0.40–3.54)
≥ 20 years	1.77 (0.22–14.2)	4.57 (2.92–7.13)

	Night shift work status in SALT sample (N = 10,366) HR (95% CI)	
	*APOE* ε4 non-carrier (n = 7226)	*APOE* ε4 carrier (n = 3140)
Night work		
No	1.00	1.00
Yes	1.16 (0.86–1.57)	1.41 (1.03–1.92)
Night work duration		
None	1.00	1.00
1–9 years	1.38 (0.94–2.03)	1.27 (0.86–1.87)
10–19 years	0.98 (0.56–1.70)	1.21 (0.67–2.19)
≥ 20 years	1.03 (0.63–1.69)	2.07 (1.25–3.44)

Model adjusted for age, sex, education, diabetes, cardiovascular disease and stroke

HR, hazard ratio; CI, confidence interval

## Discussion

In this prospective study of two cohorts, shift work and night shift work were associated with an increased risk of dementia, even when controlling for potential confounders. The association showed a slight attenuation upon adjustment for diabetes, cardiovascular disease and stroke, suggesting the association may be partly mediated by cardiometabolic disease.

Shift work of all schedule types generally predicted higher dementia rates compared to night shift work (36% compared to 12% increased rates, based on final adjusted models). This was contrary to our expectations of night shift work being associated with higher dementia incidence, based on the hypothesis that night shift work would cause a greater circadian disturbance than shift work of any type. It is possible that the observed association reflected shift work changes across time. For instance, there may have been fewer regulations surrounding shift work and shift work scheduling, i.e. poorer working and safety conditions, in the 1970 s than in later decades. One might also speculate that jobs with night shifts versus jobs with other shift work hours required greater cognitive load. Thus, worker self-selection may have masked the effects of night work. In the SALT sample, adjusting for various self-reported sleep parameters did not appear to affect the association of night work and dementia rates as we expected. However, since sleep was assessed when participants were at least 65 years of age, it is likely that the sleep item responses were not representative of sleep behavior during working life. It is also possible that the information on sleep may not be reliable as it was obtained from an older subset in which cognitive decline is prevalent; however, the SALT study population was restricted to those with no prevalent dementia or cognitive dysfunction and therefore the risk that sleep information was provided by individuals with cognitive impairment is reduced.

Longer exposure to shift work/night work appeared to be predictive of higher dementia rates. We noted that for the STR-1973 sample, spline models showed a gradual decline in hazards for dementia at approximately 20 years of shift work exposure. Rather than reflecting a true U-shaped association, we believe the dip in magnitude of association is owed to the sparsity of data available for those with 20 or more years of shift work.

Our findings are in line with those from a previous study that reported an association between ever working rotating and evening shifts and mortality from dementia in a Danish nurse cohort [9], as well as those from another prospective study observing an association between shift work and impaired cognitive performance, with the association becoming stronger for shift work durations beyond 10 years [21]. However, a recent Danish cohort study in men did not observe an association between risk of dementia and ever shift work. One possible explanation for the conflicting findings may be due to the lower life expectancy in Danes [10], particularly in men, compared to Swedes for practically all age groups and birth cohorts since the 1980s [22]. Therefore, the study population may not have survived long enough due to death from other causes [10]. Somewhat divergent from our study’s findings is a previous longitudinal study that did not find an association between shift work and normative cognitive change in late life in a sample of 595 participants [18]. It is possible that exposure to shift work may have different effects on normative age-related cognitive decline and dementia development, but mechanisms are unknown.

The association for shift work and night work duration with dementia incidence was generally more pronounced in carriers versus non-carriers of *APOE* ε4. This was more apparent in the SALT subsample, in which ever night shift work was associated with a four-fold increased risk of dementia for individuals with night shift work duration exceeding 19 years. However, given the p-value for trend was not significant, it may be that these interaction findings were spurious and hampered by the strong effect of *APOE* ε4. From the within-pair analyses, we did not see an attenuation in estimates of association between shift work/night work and dementia risk compared to those from the unpaired analyses, though it is difficult to draw conclusions based on the within-pair findings since the confidence intervals generally included a null value. Still, sleep deprivation and disturbed sleep, which are often associated with working night shifts [2], have been correlated with AD-related features such as increased brain interstitial fluid levels of amyloid beta [6] and greater amyloid burden in the brain [5]. Experimental research supports *APOE* ε4 playing a role in amyloid beta toxicity, in which ε4 carriers may exhibit reduced rates of amyloid beta clearance [23].

### Strengths and limitations

Strengths of this study are the use of two large population-based samples, long follow-up times with a median of 41 and 14 years which are appropriate for assessing dementia incidence in relation to shift work, and the adjustment for several potential confounders in models. Analyses of genotyped subsamples additionally controlled for *APOE* ε4 carrier status. In the SALT sample, individuals with baseline cognitive dysfunction were excluded from analyses to minimize reverse causality. Moreover, various sensitivity analyses were performed to rule out bias due to potential cohort effects and exposure misclassification.

Limitations concerning the measurement of the main exposures, shift work and night shift work, are acknowledged. The ever shift work and night work measures represent crude assessments of shift work and night work, but these measures are useful for comparing findings with previous studies with similar broad definitions of shift work [9, 10, 17, 21]. We could not differentiate between rotating and fixed shifts; however, an extensive review on the likelihood of permanent shift work resulting in circadian adjustment undertaken by Folkard revealed that permanent relative to rotating shift work systems did not necessarily result in substantial circadian adjustment for most individuals [24]. Nevertheless, our inability to distinguish between permanent and rotating night shift work, the regularity/irregularity of schedules, as well as the intensity, speed and direction of rotation represents a limitation of this work. Thus, the question of how these characteristics may influence incidence of dementia cannot be answered in this paper. A difference in proportion of shift workers between cohorts was noted (17.0% in STR-1973 and 30.1% in SALT) and may reflect shift work figures at the time of their respective data collection [25]. Another difference was that the SALT cohort was older on average and also had more time to be exposed to a given work schedule by the time of data collection compared to the STR-1973 cohort. Thus, since the SALT sample captures a greater proportion of workers who remained night shift workers for several years compared to the STR-1973 sample, it is possible that stronger effects of night work on the risk of dementia may have been obscured by a healthy worker survivor effect due to self-selection to prolonged night work exposure. Relatedly, since misclassification of shift work in the STR-1973 cohort analysis may have occurred due to shift work having been measured at a single time point in 1973, which represents a limitation, the effect of shift work on the risk of dementia may have been underestimated. However, sensitivity analyses taking into account shift work data from both cohorts indicates findings are robust.

Finally, while the use of national registers as a source of dementia data allows for continuous follow-up of participants’ disease and vital status, underdetection of dementia from registers may result in bias. Since only inpatient and outpatient care records were included in the NPR, dementia diagnoses performed only at the primary care level were undetected, though cases are mainly diagnosed in specialist care in Sweden [26]. However, previous validation work on Swedish register-based dementia diagnoses report moderate sensitivity of 63% and very high specificity of 99.8% [27]. Altogether, since misclassification of dementia is unlikely differential with respect to the exposure, i.e. no difference in dementia detection as a function of shift work status, any bias would be towards the null [28].

## Conclusion

In summary, findings indicate that mid-life shift work history, including night work, was significantly associated with increased incidence of dementia in later life. Furthermore, higher dementia risk was associated with long duration of shift work history. Individuals in the labor force might consider minimizing shift work exposure or managing work scheduling practices. As this is one of the first studies to report a connection between shift work and greater dementia incidence, confirmation is needed. Further work on this subject would benefit from having repeated measures of shift work, examining shift work features such as work schedule regularity, and differentiating between permanent and rotating night shift work.

## Electronic supplementary material

Below is the link to the electronic supplementary material.

Supplementary material 1 (DOCX 20 kb)
