# Supplementary material for: Shift work and risk of incident dementia: a study of two population-based cohorts
Source: Eur J Epidemiol. 2018 Aug 3;33(10):977–87. doi: 10.1007/s10654-018-0430-8 (PMC6153510; doi:10.1007/s10654-018-0430-8)
Supplement: Supplementary file 1 — Supplementary material 1 (DOCX 20 kb) [file 10654_2018_430_MOESM1_ESM.docx]

**Supplementary Materials**

**Title**: Shift Work and Risk of Incident Dementia: A Study of Two Population-based Cohorts

**Journal**: European Journal of Epidemiology

**Authors**: Kathleen Bokenberger, Arvid Sjölander, Anna K. Dahl Aslan, Ida K. Karlsson, Torbjörn Åkerstedt, Nancy L. Pedersen

**Corresponding author**:

Kathleen Bokenberger

Department of Medical Epidemiology and Biostatistics,
Karolinska Institutet, 17177 Stockholm, Sweden

Email: kathleen.bokenberger@ki.se

**Appendix A.**

Associations between shift work and incident dementia based on sensitivity analyses.

|  | Sensitivity Analysis #1  based on STR-1973 cohort | | Sensitivity Analysis #2  based on SALT cohort | | Sensitivity Analysis #3  based on SALT cohort | | Sensitivity Analysis #4  based on SALT cohort | |
| --- | --- | --- | --- | --- | --- | --- | --- | --- |
|  | N=13,283 | | N=41,199 | | N=41,199 | | N=37,140 | |
|  | HR | 95% CI | HR | 95% CI | HR | 95% CI | HR | 95% CI |
| Shift work |  |  |  |  |  |  |  |  |
| No | 1.00 | - | 1.00 | - | 1.00 | - | 1.00 | - |
| Yes | 1.18 | 1.00-1.40 | 1.13 | 1.03-1.25 | 1.13 | 1.03-1.25 | 1.13 | 0.99-1.28 |
| Shift work duration |  |  |  |  |  |  |  |  |
| None | 1.00 | - | 1.00 | - | 1.00 | - | 1.00 | - |
| 1-9 years | 1.32 | 1.09-1.60 | 1.15 | 1.01-1.30 | 1.10 | 0.97-1.25 | 1.11 | 0.95-1.31 |
| 10-19 years | 1.53 | 1.14-2.06 | 1.07 | 0.89-1.29 | 1.11 | 0.93-1.34 | 1.04 | 0.81-1.34 |
| ≥20 years | 1.12 | 0.58-2.15 | 1.15 | 0.98-1.35 | 1.19 | 1.02-1.40 | 1.21 | 0.98-1.48 |

Abbreviations: HR=hazard ratio; CI=confidence interval.

All analyses were adjusted for age, sex, education, diabetes, cardiovascular disease and stroke.

**Appendix B.**

Co-twin control analyses of association of shift work and night work with incident dementia among twins discordant for exposure and outcome in STR-1973 and SALT samples.

|  | All twin pairs | | MZ twin pairs | |
| --- | --- | --- | --- | --- |
|  | HR | 95% CI | HR | 95% CI |
|  | Shift work (STR-1973 sample) | | | |
|  | 86 discordant twin pairs | | 31 discordant MZ twin pairs | |
| No | 1.00 | - | 1.00 | - |
| Yes | 1.40 | 0.98-2.03 | 1.12 | 0.58-2.15 |
|  | Shift work duration (STR-1973 sample) | | | |
|  | 95 discordant twin pairs | | 36 discordant twin pairs | |
| None | 1.00 | - | 1.00 | - |
| 1-9 years | 1.30 | 0.86-1.96 | 0.87 | 0.42-1.80 |
| 10-19 years | 1.49 | 0.79-2.83 | 0.97 | 0.33-2.79 |
| ≥20 years | 4.94 | 1.02-23.87 | 1.21e+15 | - |
|  | Night work (SALT sample) | | | |
|  | 397 discordant twin pairs | | 59 discordant MZ twin pairs | |
| No | 1.00 | - | 1.00 | - |
| Yes | 1.28 | 1.00-1.64 | 1.01 | 0.55-1.85 |
|  | Night work duration (SALT sample) | | | |
|  | 403 discordant twin pairs | | 78 discordant MZ twin pairs | |
| None | 1.00 | - | 1.00 | - |
| 1-9 years | 1.40 | 1.02-1.92 | 0.99 | 0.50-1.95 |
| 10-19 years | 0.93 | 0.59-1.46 | 1.34 | 0.43-4.17 |
| ≥20 years | 1.39 | 0.92-2.11 | 0.93 | 0.37-2.32 |

Abbreviations: HR=hazard ratio; CI=confidence interval; MZ=monozygotic.

All analyses adjusted for age and education.
